# A simple knowledge‐based tool for stereotactic radiosurgery pre‐planning

**DOI:** 10.1002/acm2.12770

**Published:** 2019-11-19

**Authors:** Daniel S. Goldbaum, Justin D. Hurley, Russell J. Hamilton

**Affiliations:** ^1^ Department of Radiation Oncology University of Arizona Tucson AZ USA

**Keywords:** circular arc therapy (CAT), conformity index (CI), dynamic conformal arc therapy (DCAT), modeling, stereotactic radiosurgery (SRS), V12

## Abstract

We studied the dosimetry of single‐isocenter treatment plans generated to treat a solitary intracranial lesion using linac‐based stereotactic radiosurgery (SRS). A common metric for evaluating SRS plan quality is the volume of normal brain tissue irradiated by a dose of at least 12 Gy (V12), which is important because multiple studies have shown a strong correlation between V12 and incidence of radiation necrosis. Unrealistic expectations for values of V12 can lead to wasted planning time. We present a model that estimates V12 without having to construct a full treatment plan. This model was derived by retrospectively analyzing 50 SRS treatment plans, each clinically approved for delivery using circular collimator cone arc therapy (CAT). Each case was re‐planned for delivery via dynamic conformal arc therapy (DCAT), and then scaling arguments were used to extend dosimetric data to account for different prescription dose (PD) values (15, 18, 21, or 24 Gy). We determined a phenomenological expression for the total volume receiving at least 12 Gy (TV12) as a function of both planning target volume (PTV) and PD: $\left[TV12/\left(1cc\right)\right]=\left\{n*\left[PD/\left(1Gy\right)\right]+d\right\}*{\left[PTV/\left(1cc\right)\right]}^{a*{\left[PD/\left(1Gy\right)\right]}^{c}}$, where $\left\{a,c,n,d\right\}$ are fit parameters, and a separate set of values is determined for each plan type. In addition, we generated a sequence of plots to clarify how the relationship between conformity index (CI) and TV12 depends on plan type (CAT vs DCAT), PTV, and PD. These results can be used to suggest realistic plan parameters and planning goals before the start of treatment planning. In the absence of access to more sophisticated pre‐planning tools, this model can be locally generated and implemented at relatively low cost with respect to time, money, and expertise.

## 1 INTRODUCTION

Stereotactic radiosurgery (SRS) is a course of radiotherapy delivered in a single high‐dose fraction with submillimeter spatial accuracy, and steep dose fall‐off outside the target volume.^1^ SRS is mostly used to treat intracranial lesions, which can be either malignant, such as brain metastases, recurrent glioma, or vestibular schwannoma; or benign, such as meningiomas or arteriovenous malformations. SRS can be delivered using several modalities, of which the most commonly used are Leksell Gamma Knife (GK; Elekta Instrument AB, Sweden), Cyberknife (CK; Accuray, Sunnyvale, California), and more conventional radiotherapy linear accelerators outfitted to deliver stereotactic radiosurgery (linac‐based SRS).^1^, ^2^, ^3^, ^4^ In this study, we analyze the dosimetry of single‐isocenter treatment plans generated to treat a solitary intracranial lesion using linac‐based SRS.

Before the start of computer‐based treatment design and dose calculation, the dosimetric task is specified by a radiation oncologist choosing plan features, goals, and constraints. We define “pre‐planning” as the process by which these choices are made. In our clinic, a radiation oncologist determines the gross tumor volume (GTV), planning target volume (PTV), prescription dose (PD), dose restrictions for organs at risk (OAR), fractionation, dose delivery method, and goals for plan conformality and coverage. For intracranial SRS, an especially important dose restriction metric is the volume of normal brain tissue irradiated with at least 12 Gy (V12). Multiple retrospective studies have shown a strong correlation between V12 and the incidence of radiation necrosis.^5^, ^6^, ^7^ In particular, Minniti et al.^6^ presented the correlation in quartiles, each characterized by a single actuarial risk of necrosis for a given range of V12. Thus, the values of V12 separating adjacent risk quartiles are attractive planning goals to maximize dose delivered to the target while minimizing the risk of necrosis. Since a V12 goal represents the allowable risk of necrosis, a plan that fails to meet this goal is likely to be rejected. Additionally, if the corresponding pre‐planning process has specified a dosimetric task with unattainable goals, then treatment planning time has been wasted, and disturbances in clinical workflow and treatment scheduling can follow.

To help preclude this outcome, we present a model that predicts V12 as a function of PTV, GTV, PD, and radiation delivery type. While accurate target delineation fixes the GTV, the PTV, PD, and radiation delivery type can be adjusted based on case‐specific factors including the difference between desired and predicted values of V12. The PTV is generated from the GTV by adding a margin to account for sources of uncertainty, or in some clinics no margin. V12 can be reduced by reducing the margin, but this increases the risk of geometric miss. The predicted value of V12 is also reduced by lowering the PD, as well as by the correct choice of treatment delivery method. These ideas are further developed and quantified in the results and discussion sections below.

In our clinic, initial PD values were chosen based (roughly) on the guidelines set by Radiation Therapy Oncology Group (RTOG) 90‐05 for SRS treatment of “recurrent, previously irradiated metastases,”^8^ and by RTOG protocol 1270 guidelines for treatment of unresected brain metastases.^9^ RTOG protocol 90‐05 determined the maximum tolerated doses to be 24, 18, and 15 Gy for tumors with maximal diameter of ≤20, 21–30, and 31–40 mm, respectively.^8^ RTOG protocol 1270 guidelines state that the maximal cross‐sectional diameter for the GTV must be <30 mm; and the prescription dose shall be 24, 22, and 20 Gy for lesions of maximal diameter <10 mm, ≥10 but <20 mm, and ≥20 but <30 mm, respectively.^9^ A PD range of 18–24 Gy is most commonly used, though a retrospective review published in 2016 suggests that for tumors with maximal diameter ≤20 mm a PD of 24 Gy is correlated with improved local control without increased incidence of radiation necrosis.^10^ The local prescribing convention in our clinic is at the conservative end of the above standards. That is, we do not treat patients with SRS if the maximal target diameter is >20 mm, and the maximum target diameter considered in this study is 18.3 mm.Several treatment delivery methods are available using linac‐based SRS systems. These include circular arc therapy (CAT), dynamic conformal arc therapy (DCAT), intensity modulated radiosurgery (IMRS), and volumetric modulated arc therapy (VMAT).^2^, ^11^, ^12^, ^13^, ^14^ CAT delivers radiation via non‐coplanar circular arcs collimated by stereotactic cone attachments with circular apertures that vary in size from 4 to 50 mm. DCAT delivers radiation by non‐coplanar circular arcs collimated by micro‐multileaf collimators (mMLCs) so that the resulting aperture conforms to the beam's eye view (BEV) of the target at control points along the arc. IMRS is the familiar inverse‐planned intensity modulated radiation therapy (IMRT) delivered in a single fraction at fixed gantry positions. VMAT delivery is like IMRS, except that treatment is delivered via non‐coplanar arcs where the dose rate and gantry rotation rate can change independently.^12^, ^13^, ^14^ When choosing a treatment delivery method, one should consider the differences in dosimetric coverage and conformality that are characteristic of the corresponding dose distributions.

At our clinic, treatment plan dosimetry is evaluated by several metrics, including those representing the target coverage by the prescription dose and the conformality of the treated volume to the PTV. The coverage (COV) denotes the percentage of the PTV receiving the PD or greater. The RTOG 90‐05 defined conformity index $CI=\left(PIV/PTV\right)$, where PIV (prescription isodose volume) is the volume receiving at least the PD.^8^ In our clinic, plan evaluation via both COV and CI is preferred to plan evaluation via a single metric such as the Paddick conformity index (PCI), which takes both conformality and coverage into account.^15^ CAT delivery has the smallest penumbra, resulting in plans with the steepest dose fall‐off.^11^ However, the CAT collimation aperture is always circular, and thus the shape of the prescription isodose surface for a single isocenter usually resembles an ellipsoid.^16^, ^17^ When the target does not resemble an ellipsoid, the mMLC system of DCAT can achieve smaller CI when compared to the CAT plan,^11^ but with a smaller gradient of dose fall‐off outside the target, which can lead to unexpected differences in the relative values of V12. While arc delivery is standard for linac‐based SRS systems, there is evidence that IMRS can be a valid alternative to DCAT or CAT, even though fixed beam delivery can lead to increased values of entrance and exit dose along the beam axes.^11^, ^18^ In comparison, VMAT plans are advantageous because they combine arc delivery with intensity modulation to achieve less low‐dose coverage than with IMRS, and greater target conformity than that of DCAT.^13^ In addition, state‐of‐the‐art VMAT systems can now treat multiple targets with a set of non‐coplanar arcs associated with a single isocenter, which can drastically reduce treatment time.^12^, ^13^, ^14^ The correct choice of delivery method can help produce an optimal treatment plan while also reducing treatment planning time.

In this study, we retrospectively analyze CAT plans that were delivered in our clinic between 11/19/2010 and 8/6/2016, the associated DCAT re‐plans, and further data generated for different PD values. During this time, our linac‐based SRS system did not have the capability to accurately deliver IMRS plans to such small targets (in this study, the largest maximal target diameter was 18.3 mm), or to deliver VMAT plans at all. Although we do not address IMRS or VMAT, the results of this study can benefit the large number of clinics that do not yet use these methods for treating small intracranial lesions and also centers with VMAT. A recent study generated a predictive model of V12 for single‐target single‐isocenter SRS delivery using only DCAT plan data, and showed that it could be accurately applied to dosimetric prediction of multi‐target single‐isocenter VMAT plans.^19^ This suggests that a similar use could be feasible for the model generated in the current study.

There are several pre‐planning decisions that affect V12, and thus the risk of radiation necrosis. During pre‐planning, a reasonable expectation for V12 as a function of the GTV, PTV, PD, and linac‐based SRS delivery method can inform such decisions and increase the likelihood that an acceptable plan can be achieved with relative ease and speed, and without the need for re‐plans that can delay the start of treatment and disrupt clinical workflow. The current study demonstrates how to generate and implement such a predictive model for V12. The process is shown to be relatively low cost with respect to time, money, and expertise. Although it is reasonable to expect V12 to be related to the dosimetrist, target geometry, and CI, we construct a simplified model without parameters to account for these features. Instead, our model depends only on PD and PTV, but still provides a reliable estimate of V12, as evidenced by the fact that the minimum coefficient of determination from the data fitting presented in this paper is 0.921. Additionally, even though the model does not explicitly depend on CI, we present a set of plots to demonstrate the relationship between CI and V12 values from CAT and DCAT plans generated for the same PTV.

## 2 MATERIALS AND METHODS

### 2.1 General data characteristics

A retrospective study with institutional review board approval was performed using 50 single‐isocenter SRS treatment plans, each delivered in the department of radiation oncology at the Banner University Medical Center in Tucson, AZ. The plans were each generated and analyzed using the BrainScan 5.32 treatment planning system (TPS) (BrainLab AG, Germany), which was commissioned for use with the department's Novalis linac‐based SRS system equipped with 6 MV photon beam, 800 MU/min maximum dose rate, stereotactic cone mount, m3 micro multileaf collimator (mMLC), and the ExacTrac patient positioning system. The plans were approved for treatment delivery between 11/19/2010 and 8/6/2016 and were generated by multiple dosimetrists.

In each case, the department's Philips Brilliance Big Bore CT Simulator (Philips, The Netherlands) was used to generate a planning scan, which was imported to BrainScan. Then, the planning scan was usually fused to one or more additional imaging sets (T1‐ and T2‐weighted MRI, with or without contrast). Next, the GTV was contoured by a radiation oncologist, who also generated the PTV by adding a margin of 0–2 mm, or by manual contouring. OARs, including the brainstem, eyes, optic nerves, chiasm, and normal brain tissue and when necessary the cochleae, were also contoured. To facilitate a plan with acceptable coverage, conformality, and steep dose drop‐off outside the target, the prescription isodose line (PIDL) was set to an initial value of 80%, but this value could be adjusted during treatment planning.^16^, ^17^ In our department, the nominal SRS planning goals were COV > 99%, CI < 1.5, and V12 < 3.3 cc. But these values could be modified to account for patient‐specific factors.

### 2.2 Treatment planning

#### 2.2.1 Treatment planning for CAT

For CAT plans, beam collimation was achieved using stereotactic cones with available circular aperture diameters of 4, 6, 7.5, 10, 12.5, 15, 17.5, and 20 mm. The dose distribution is calculated in the planning system algorithm by first modeling each arc as an evenly spaced, isocentric array of fixed beams. Then, the dose contribution of each fixed beam was calculated using a pencil beam algorithm, where density inhomogeneity of the imported CT scan used for treatment planning was taken into account by a standard radiological path length (RPL) correction. For each voxel of that scan, the calculated dose contribution from a single arc was the sum of the calculated dose contribution from each of that arc's constituent fixed beams, and the calculated dose for the whole treatment plan was then the sum of the calculated dose delivered by each arc.^20^

Treatment planning was performed starting with a standard template for delivery of radiotherapy via arcs. Each arc corresponds to a table angle while the angular extent of that arc corresponds to the angular extent of gantry rotation at a fixed table angle. Delivery of a single arc for SRS is achieved via gantry rotation about a fixed orientation of the treatment couch (couch angle). For SRS of brain metastases, the template consisted of six arcs delivered at couch angles of 0°, 30°, 60°, 270°, 300°, and 330°; each with a gantry rotation from 40 to 140 or from 320° to 220°; and with beam collimation for each arc via the same stereotactic cone. If necessary, the plan was further optimized by adjusting the number, length, orientation, and relative weighting of the arcs; the size and variety of the stereotactic cones; and the location of, and dose delivered to, the isocenter.^16^, ^17^

#### 2.2.2 Re‐planning for DCAT

Each of the 50 CAT plans were re‐planned for delivery via DCAT. To achieve a meaningful comparison between CAT and DCAT plans, each DCAT re‐plan was performed using the following planning goals with respect to the corresponding CAT plan: COV within ±0.5%, lower CI, and maximum dose values to OARs ≤ [(CAT value) + 1] Gy. These goals were met, except for three cases where the CI increased, one case where the CI was unchanged; and one case where the maximum dose to an OAR increased by 1.1 Gy. It would have been optimal to fix the DCAT plan COV value to that of the corresponding CAT plan, but with the forward planning approach to DCAT, this was very difficult to achieve. The ±0.5% goal was achievable, and for a given patient, DCAT plans with different COV values within that range showed insignificant changes to the corresponding TV12 values.

For DCAT plans, beam collimation was achieved using the m3 mMLC system, which allows a maximum field size of 9.8 × 9.8 cm^2^ at isocenter and is made up of 26 leaf pairs. The number and width of leaf pairs, from innermost (central) to outermost, is 14 × 3.0 mm, 6 × 4.5 mm, and 6 × 5.5 mm. For dose calculation in the planning system, each arc was characterized by N control points, each consisting of an MLC and jaw configuration specified to conform to the BEV of the target or adjusted to optimize dose coverage and conformality. For each voxel of the treatment planning scan, the dose contribution from a DCAT arc was calculated by summing the dose contributions from N‐1 “arc segment beams” each located at the midpoint of the arc segment connecting adjacent control points, and taking into account the continuous beam delivery and leaf motion between control points.^20^ The total planned dose for each voxel was the sum of the dose contributions from each arc.

Treatment planning for delivery via DCAT was performed beginning with nearly the same template as used for CAT planning. The only difference is that beam collimation in the DCAT plan is achieved via configurations of the MLCs, instead of with stereotactic circular aperture cones. The MLC configuration is determined by automatically conforming to an additional planning volume (“shaper”) that is generated by automated expansion of the planning target volume. The optimization procedure for DCAT plans is similar to that for CAT plans, except instead of adjusting the beam collimation by changing the stereotactic cones, the planner adjusts the shaper to which the MLCs conform.

### 2.3 Analysis of plan data

#### 2.3.1 Data collection, generation, and specification

For each plan, information on both the geometry of the lesion, and dosimetry of the treatment plan were collected using the TPS. For each lesion, the GTV, PTV, and in analogy with an ellipsoid, the diameter (boundary‐to‐boundary distance) of each “principle axis” of the PTV was recorded. The diameters were determined by centering the intersection point of the sagittal, coronal and transverse viewing planes on the isocenter; then measuring (a) the longest PTV diameter intersecting the isocenter; (b) in the same viewing plane the diameter perpendicular to that longest diameter; and (c) the diameter perpendicular to that viewing plane. For each plan, the COV, PTVMD (PTV maximum dose; isodose percentages refer to this value), PIV (volume enclosed by the prescription isodose surface), TV50% (volume enclosed by the 50% isodose surface), and TV12 (volume enclosed by the 12 Gy isodose surface) were recorded, and then used to determine the PIDL, CI, and V12.

There are two distinct cases for determination of V12 for which the volume of irradiated brain parenchyma is the issue. In this study, the volume enclosed by the inner surface of the skull is recognized as brain parenchyma. When the 12 Gy isodose surface is entirely within the brain parenchyma, the enclosed volume is the TV12 defined above, and V12 = TV12‐GTV. But in the case where the 12 Gy isodose surface extends beyond the brain parenchyma, then only the enclosed parenchyma volume (including the GTV) is counted. We call this volume the clinical TV12 (cTV12) so that V12 = cTV12‐GTV. Since our objective is to generate a simple model for V12 based on the geometry of the target, the cTV12 data are too specific to be useful. For this reason, we perform our analysis using only TV12 for each plan regardless of whether the 12 Gy isodose surface includes only brain parenchyma, or not. As a result, our analysis cannot be directly applied to plans where it is obvious *a priori* that V12 must be determined from cTV12 but can be used in conjunction with a case‐specific estimate of TV12‐cTV12. The above definitions are consistent with the convention used by Minniti et al.^6^

In this study, each treatment plan is characterized by a set of dosimetric parameter values. To facilitate direct inter‐plan comparison between like parameters, we generate additional data by rescaling the prescription doses on our clinically treated CAT plans, and the corresponding DCAT plans. The change of PD does not change the relative spatial dose distribution, but only scales the absolute dose value at each point in space by the same factor as that of the PD change. It follows that the COV, PIDL, CI, and IDLs all remain the same. So, to determine the values of TV12 for the different PDs, we only need to consider the volume enclosed by the appropriate isodose surface. For example, when PD = 24 Gy, then TV12 = TV50%, and when PD = 15 Gy, then TV12 = TV80%, etc. The result, without any further calculation, is a total of 400 sets of dosimetric data: 50 sets for each combination of plan type (DCAT or CAT) and PD (15, 18, 21, or 24 Gy).

#### 2.3.2 Model of TV12 as a function of PTV and PD

Plotting and data fitting were performed using MATLAB 2016b (MathWorks; Natik, MA). The data were organized into eight categories characterized by plan type (DCAT or CAT), and PD (15, 18, 21, or 24 Gy). Each category consisted of 50 sets of dosimetric data, each set corresponding to a treatment plan. For each category, $\log\left[TV12/\left(1cc\right)\right]$ vs $\log\left[PTV/\left(1cc\right)\right]$ was plotted together with the corresponding fit to a first‐order polynomial. In this case, “log[]” implied “log_10_[],” and each volume value was divided by “1 cc” so that for each point the argument was dimensionless. In this way, the TV12 vs PTV relationship within each data category was characterized by two dimensionless fit parameters: slope (m) and intercept (b). For each plan type (CAT or DCAT), we plotted and fit both (m vs PD) and (b vs PD) data sets. Combining the data fit functions and parameter values determined during each round of data fitting, we generated a relationship for TV12 as a function of both PTV and PD.

#### 2.3.3 Comparison of conformity and low‐dose coverage between CAT and DCAT plans

First, we organize the data into four groups on the basis of PD value (15, 18, 21, and 24 Gy), where each group consists of dosimetric data from 50 CAT plans, and from the corresponding 50 DCAT re‐plans. Then, a comparison of conformity and low‐dose coverage between CAT and DCAT treatment plans was performed by first calculating the change in CI and TV12 values between the CAT plan and the DCAT re‐plan $\left(\Delta CI,\Delta TV12\right)$. Then, within each data group, the corresponding ΔCI and ΔTV12 values were plotted against PTV.

## 3 RESULTS

### 3.1 Model of TV12 vs PTV and PD

**Figure 1:**
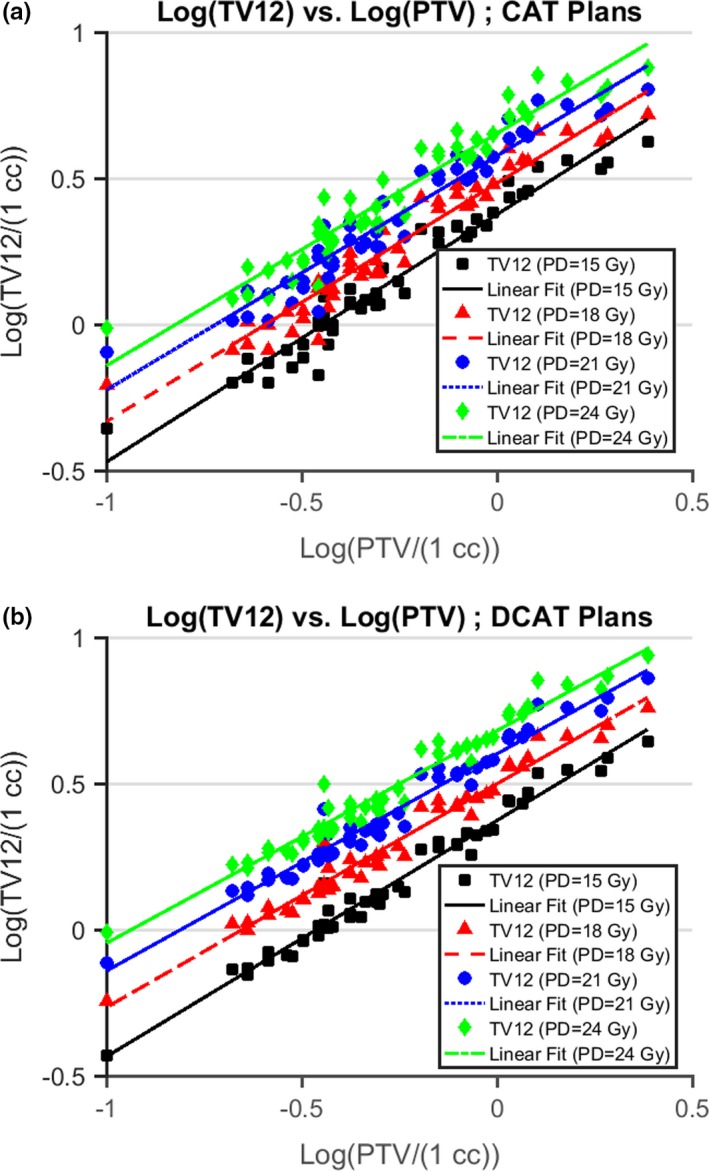
Fit of TV12 vs planning target volume (PTV). For each plan type [circular arc therapy (CAT) or dynamic conformal arc therapy (DCAT)] and each prescription dose (PD = 15, 18, 21, or 24 Gy), the relationship between volume receiving dose of at least 12 Gy (TV12) and PTV is modeled by linear fit of $\log_{10}\left[TV12/\left(1\;cc\right)\right]$ vs $\log_{10}\left[PTV/\left(1\;cc\right)\right]$. For CAT (DCAT) plans, the minimum value of the coefficient of determination is 0.921 (0.962).

For each data category, we plotted $\log\left[TV12/\left(1\;cc\right)\right]$ vs $\log\left[PTV/\left(1\;cc\right)\right]$, and fit the data to

(1) $$\log\left[TV12/\left(1\;cc\right)\right]=m*\log\left[PTV/\left(1\;cc\right)\right]+b,$$

where *m* (slope) and b (intercept) are unitless parameters. The form for Eq. (1) was chosen because for each energy a linear plot of TV12 vs PTV displayed an exponential relationship. So using logarithms is preferable since analysis of multiple data sets is much easier when the data in each set are linearly related. The raw data (in logarithmic form) and lines of best fit are displayed in Fig. 1 while the corresponding parameter values are found in Table 1. For clinical use, we plot TV12 vs PTV by solving Eq. (1) for $\left[TV12/\left(1\;cc\right)\right]$.

(2) $$\left[TV12/\left(1\;cc\right)\right]=10^{b}*{\left[PTV/\left(1\;cc\right)\right]}^{m}.$$

**Table 1:** $\log_{10}\left[TV12/\left(1\;cc\right)\right]$ vs $\log_{10}\left[PTV/\left(1\;cc\right)\right]$ — linear best fit parameter values.

Prescription dose (Gy)	Slope, m		Intercept, b		Coefficient of determination, R^2^	
	CAT	DCAT	CAT	DCAT	CAT	DCAT
15	0.847	0.808	0.376	0.373	0.947	0.974
18	0.816	0.765	0.483	0.499	0.935	0.969
21	0.801	0.743	0.576	0.601	0.926	0.965
24	0.795	0.727	0.654	0.680	0.921	0.962

For each data category specified by plan type [circular arc therapy (CAT) or dynamic conformal arc therapy (DCAT)] and prescription dose (PD = 15, 18, 21, or 24 Gy), the relationship between total volume enclosed by the 12 Gy isodose surface (TV12) and the planning target volume (PTV) was determined by a linear fit of $\log_{10}\left[TV12/\left(1\;cc\right)\right]$ vs $\log_{10}\left[PTV/\left(1\;cc\right)\right]$. Each fit was characterized by the values of slope (m), intercept (b), and coefficient of determination ($R^{2}$).

**Figure 2:**
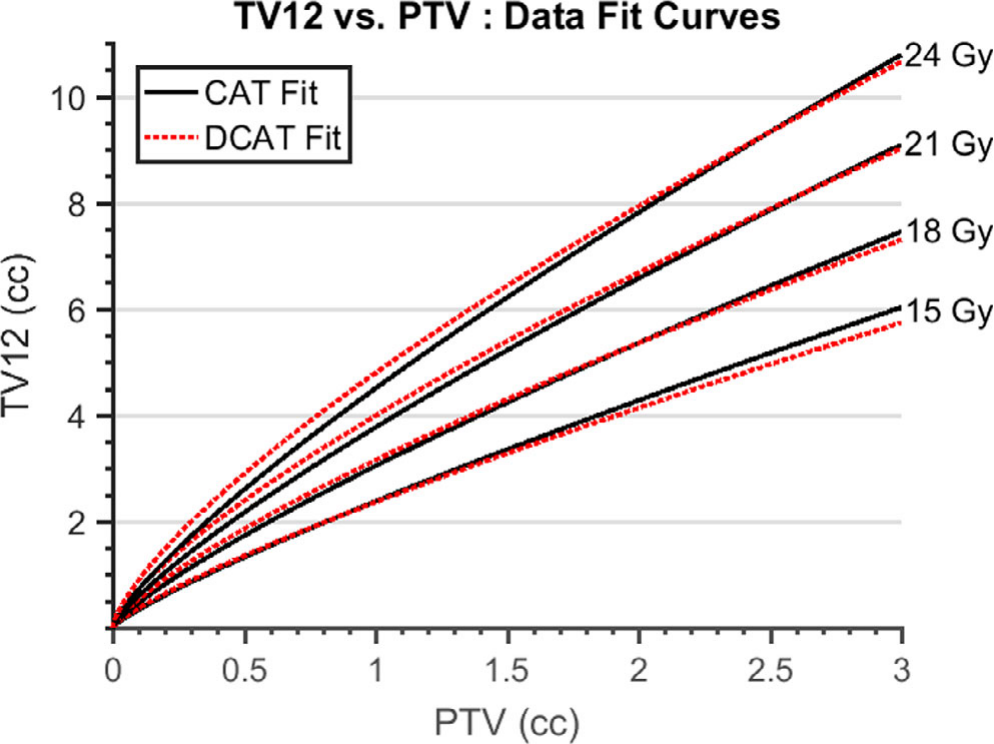
TV12 vs planning target volume (PTV): data fit curves. Fit curves for total volume receiving at least 12 Gy (TV12) as a function of the PTV. A fit curve was determined for each of eight data categories characterized by prescription doses (PD) of 15, 18, 21, or 24 Gy; and either circular arc therapy (CAT) or dynamic conformal arc therapy (DCAT) plan types. This constraint allows us to analyze the difference in TV12 due to the difference in treatment delivery method. Compared to the CAT TV12, the DCAT TV12 is larger (smaller) for relatively small (large) PTV and greater (lesser) PD. A radiation oncologist can use a plot such as this as a rough guide to determine if SRS treatment is feasible, and if so, to choose the amount of margin added to the gross tumor volume (GTV), the PD, and the SRS delivery method to meet the desired volume of normal tissue receiving at least 12 Gy (V12 = TV12 − GTV).

The fit curves for each data category are plotted together in Fig. 2.

**Figure 3:**
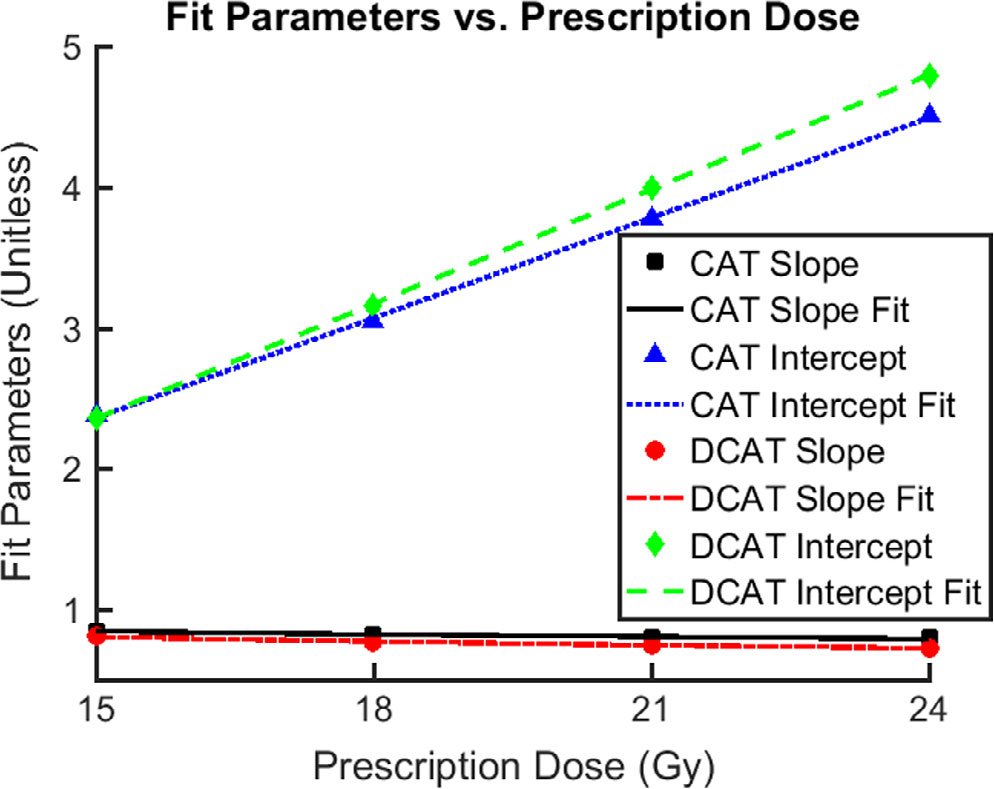
Fit parameters vs prescription dose. For each of the eight data categories corresponding to plan type [circular arc therapy (CAT) or dynamic conformal arc therapy (DCAT)] and (PD = 15, 18, 21, or 24 Gy), the parameters that characterize each category's fit to $\log_{10}\left[TV12/\left(1cc\right)\right]=m*\log_{10}\left[PTV/\left(1cc\right)\right]+b$ are plotted against PD. The parameters are organized into four groups on the basis of plan type (CAT or DCAT) and parameter type (slope (m) or intercept (b)). For each plan type, the set of slope parameters is fit to $m=\text{a*}\;{\left[\text{PD}/\left(1\;\text{Gy}\right)\right]}^{c}$ and the set of intercept parameters is fit to $10^{b}=\text{n*}\left[\text{PD}/\left(1\;\text{Gy}\right)\right]+d$. For CAT (DCAT) plans, the coefficient of determination ($R^{2}$) is 0.946 (0.978) for the slope fit, and 0.9995 (0.9999) for the intercept fit.

For each plan type, the PD dependence is determined by fitting m vs PD to

(3) $$m=a*{\left[PD/\left(1\;Gy\right)\right]}^{c},$$

and b vs PD to

(4) $$10^{b}=n*\left[PD/\left(1\;Gy\right)\right]+d,$$

where each fit is characterized by the unitless coefficient (*a*), exponent (*c*), fit slope (*n*), and fit intercept (*d*). The plots of m vs PD, b vs PD, and the corresponding fit curves are displayed in Fig. 3. The values determined for a, c, n, and d are found in Table 2. An expression for TV12 is found by substituting Eqs. (3) and (4) into Eq. (2):

(5) $$\left[TV12/\left(1\;cc\right)\right]=\left\{n*\left[PD/\left(1\;Gy\right)\right]+d\right\}*{\left[PTV/\left(1\;cc\right)\right]}^{a*{\left[PD/\left(1\;Gy\right)\right]}^{c}}.$$

**Table 2:** (PTV, TV12) — fit parameters vs prescription dose.

Unitless parameters	$m=a^{*}{\left[\text{PD}/\left(1\;\text{Gy}\right)\right]}^{c}$			$10^{b}=n^{*}\left[\text{PD}/\left(1\;\text{Gy}\right)\right]+d$		
	a	c	$R^{2}$	n	d	$R^{2}$
CAT	1.222	−0.137	0.946	0.237	−1.195	0.9995
DCAT	1.481	−0.226	0.978	0.271	−1.708	0.9999

Each category of (PTV, TV12) data was characterized by separate set of fit parameters: slope (m) and intercept (b). The relationship of m and b to the prescription dose (PD) was then fit to $m=a^{*}{\left[\text{PD}/\left(1\;\text{Gy}\right)\right]}^{c}$ and $10^{b}=n^{*}\left[\text{PD}/\left(1\;\text{Gy}\right)\right]+d$, respectively. This table displays the (unitless) values of the fit parameters $\left(a,c,n,d\right)$, as well as each corresponding coefficient of variation, $R^{2}$. Parameter values are specific to plan type, either circular arc therapy (CAT) or dynamic conformal arc therapy (DCAT).

**Figure 4:**
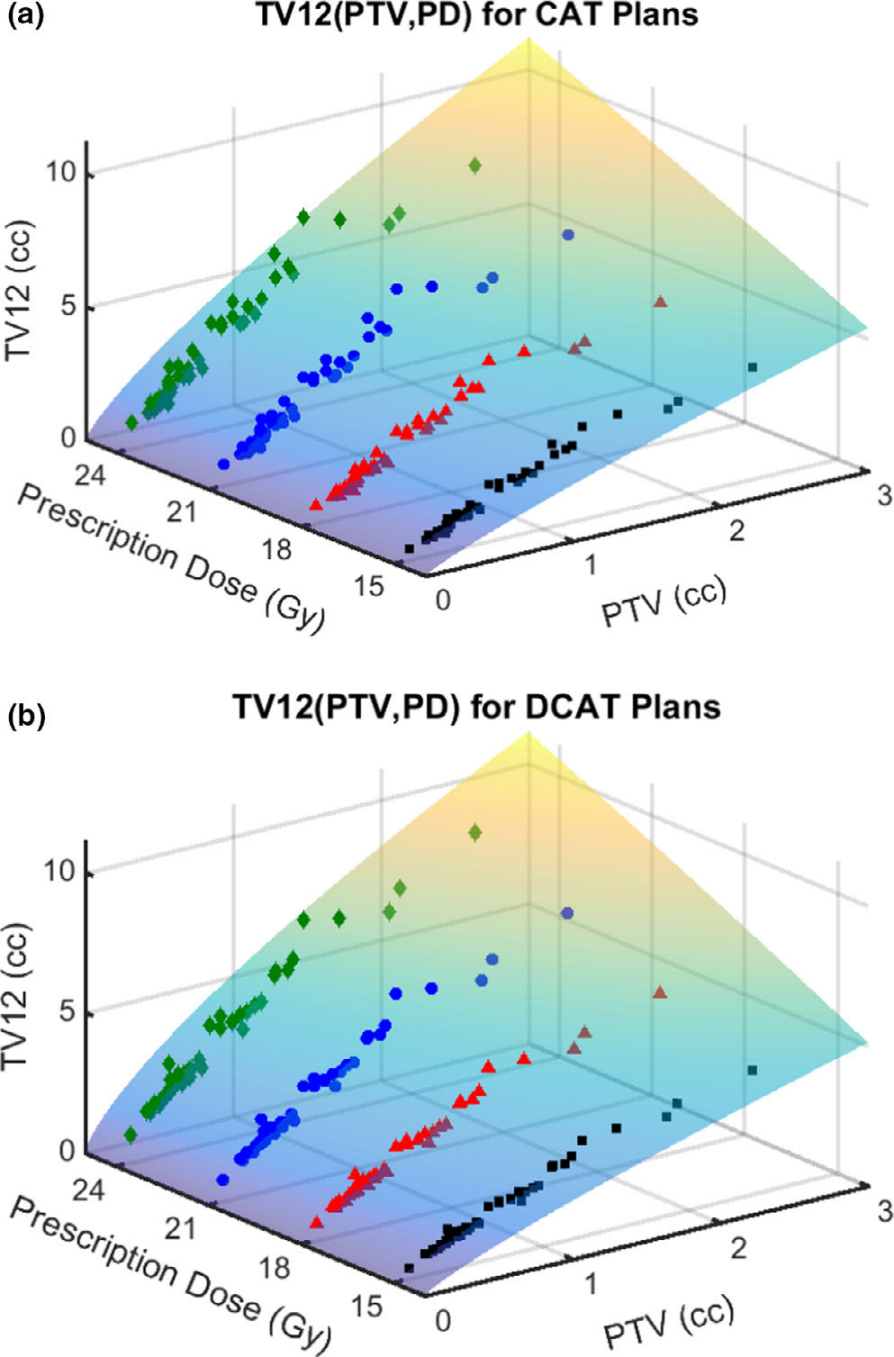
TV12 data fit as a function of planning target volume (PTV) and prescription dose (PD). Separate plots were generated for (a) circular arc therapy (CAT) and (b) dynamic conformal arc therapy (DCAT) plan types. Each plot displays a smooth function (with surface colored to reflect magnitude) fitting total volume receiving at least 12 Gy (TV12) as a function of both PTV and PD. The surface functions are of the form $\left[TV12/\left(1cc\right)\right]=\left\{n*\left[PD/\left(1Gy\right)\right]+d\right\}*{\left[PTV/\left(1cc\right)\right]}^{a*{\left[PD/\left(1Gy\right)\right]}^{c}}$, with a specific set of parameter values for each plan type. Each surface is plotted together with the corresponding four TV12 vs PTV data sets characterized by PD values of 15, 18, 21, or 24 Gy for which each data point is displayed as a black box, red triangle, blue circle, or green diamond, respectively. The agreement between the smooth curve and the raw data sets can be visualized since the points below the surface are darker than those above the surface. The model equation is expected to be generally applicable while the parameter values are specific to the clinical service from which the data were collected. In a clinical setting, the surface functions, with corresponding parameter values, can be used to quickly determine a rough value of the expected TV12 for a given PTV and the PD.

For each plan type, Eq. (5) with the corresponding set of parameter values is plotted together with the corresponding raw data sets {(PTV, TV12); PD = 15, 18, 21, or 24 Gy} in Fig. 4. The expected value of V12 is readily determined by subtracting $\left[GTV/\left(1\;cc\right)\right]$ from Eq. (5).

### 3.2 Comparison of conformity and low‐dose coverage between CAT and DCAT plans

**Figure 5:**
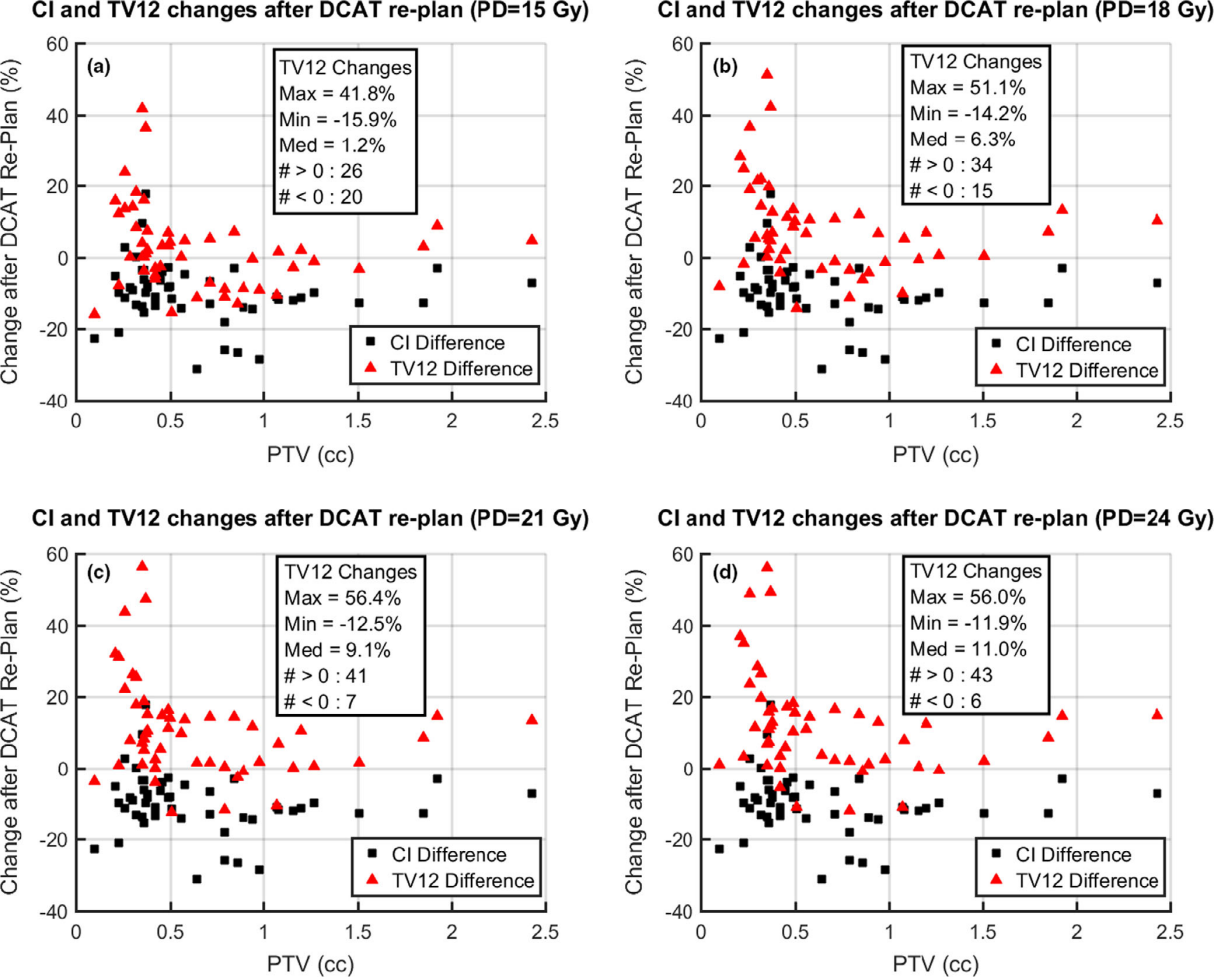
Comparison of dynamic conformal arc therapy (DCAT) and circular arc therapy (CAT): CI and TV12. Fifty clinically approved CAT treatment plans were re‐planned for delivery via DCAT. For each plan, the total volume receiving at least 12 Gy (TV12) and the conformity index (CI) is recorded. Additional data were generated using the invariance of the relative dose profile with respect to change of the prescription dose (PD). For each CAT/DCAT re‐plan pair, the percent change in both TV12 and CI are plotted vs PTV in subfigures (a) PD = 15 Gy, (b) PD = 18 Gy, (c) PD = 21 Gy, and (d) PD = 24 Gy. Due to the aforementioned scaling property of the dose distribution, the set of CI values are identical for each prescription dose. But as the PD increases, more of the TV12 values increase, as does the median change in TV12.

For each fixed PD data set, ΔCI and ΔTV12 are plotted against the corresponding PTV (Fig. 5). ΔCI is the percent difference defined by

(6) $$\Delta CI\equiv \frac{\left(CI_{\text{DCAT}}-CI_{\text{CAT}}\right)}{CI_{\text{CAT}}}\times 100\%,$$

where $CI_{\text{DCAT}}$ and $CI_{\text{CAT}}$ are the conformity index values for the corresponding DCAT and CAT treatment plans, respectively. ΔTV12 is the percent difference defined by

(7) $$\Delta TV12\equiv \frac{\left(TV12_{\text{DCAT}}-TV12_{\text{CAT}}\right)}{TV12_{\text{CAT}}}\times 100\%,$$

where $TV12_{\text{DCAT}}$ and $TV12_{\text{CAT}}$ were the values for the corresponding DCAT and CAT treatment plans, respectively. For each PD, the set of ΔTV12 changes is characterized by the maximum, minimum, and median values; as well as by the number of increasing and decreasing values. Each set of values is tabulated within the corresponding subplot of Fig. 5.

## 4 DISCUSSION

We have presented a model of TV12 as a function of PTV, PD, and treatment delivery type (CAT or DCAT). The model is independent of CI, but we have analyzed the difference in CI (ΔCI) and in TV12 (ΔTV12) observed between CAT and DCAT plans, and how these differences depend on PD.

### 4.1 TV12 modeling

The TV12 model is significant because it allows a pre‐plan forecast of V12, which has been shown to be strongly correlated to the post‐SRS incidence of radiation necrosis.^6^, ^7^ Pre‐plan forecasting of V12 assists the prescribing physician in choosing plan type, PD, and margin size that correspond with acceptable and realistic goals for V12. This knowledge‐based approach to pre‐planning lessens the likelihood that re‐planning will be necessary, thus forestalling a delay to the start of treatment while improving clinical workflow. Although the model has the greatest predictive power when it is generated from local clinical data (roughly analogous to the commissioning of a treatment planning system), the cost for setup and implementation (measured in time, expertise, and money spent) is relatively small compared to more sophisticated knowledge‐based tools.^21^ In an ideal situation, every clinic would have the resources to acquire such powerful tools, but for situations where this is not viable the current study shows how an effective surrogate can be generated and implemented.

After data have been acquired, the first part of the modeling procedure is to plot and fit TV12 vs PTV, for each of the eight data categories specified by PD (15, 18, 21, or 24 Gy) and plan type (CAT or DCAT). First, we note that our results are presented as functions of PTV, instead of maximum tumor diameter as commonly used to set SRS prescriptions. This is because hypothesis of ours was that the TV12 would be better correlated with PTV than the linear dimension since the volume irradiated is constructed to be as conformal as possible to the PTV. The results of the model fit support this hypothesis. Second, one might ask why not plot V12 vs GTV, since V12 is the quantity correlated to incidence of radiation necrosis and GTV is the visible extent of the lesion? We choose to plot TV12 vs PTV because these quantities allow us to best separate the tasks performed by the dosimetrist from those performed by the prescribing physician. That is, the physician specifies the PD, the PTV (by adding margin to the GTV), the plan type (sometimes in consultation with the dosimetrist and/or physicist), OAR dose restrictions, and any other dosimetric goals. If we then focus on the V12 goal, the task of the dosimetrist can be distilled to satisfying the other stated dosimetric goals while minimizing TV12 for the given PTV. (Though an exception can occur in the case where the 12 Gy isodose surface is not enclosed within the brain parenchyma, and thus an increase in TV12 does not necessarily imply the same increase in V12.) Using the TV12 model, the physician can quickly estimate V12 (=TV12 – GTV) and can then modify pre‐planning details accordingly.

One characteristic of the TV12 vs PTV data analysis is that the fits for CAT plan categories (R^2^ = 0.921–0.947) are worse than the fits for the DCAT plan categories (R^2^ = 0.962–0.974). This is somewhat surprising given that the DCAT plans were constructed to be comparable to the original CAT plans based on COV and OAR doses. However, as will be discussed in section IV.B, these parameter values alone do not completely specify the dose distribution of the plan. The subtle differences in the dose distribution and planning methodology between CAT and DCAT plan types, combined with the fact that the DCAT plans were all generated by the same individual (DSG), may be the reason the model fits DCAT better. Even so, the CAT fits are sufficient to provide useful TV12 estimates, which suggests that the model is robust to the data variation introduced by multiple dosimetrists. Such robust character is desirable for clinical use, where one cannot place strict controls on target conditions and planning dosimetry.

Another notable feature of the TV12 vs PTV data is that in some cases, a group of plans with closely spaced PTV values produced a much wider range of TV12 values. An attempt was made to determine a pre‐planning parameter, to be used in place of the PTV, which would better differentiate between these cases and thus add to the predictive power of the model. Our hypothesis was that an increase in TV12 could be related to an increase in the surface area of the target, and the surface area of the target could be quantified by analogy with an ellipsoid. For ellipsoids of identical volume, the surface area depends on the diameters measured along the principle axes. For each target, we recorded these diameters, and then calculated the surface area for an ellipsoid with the same volume (PTV) and ratios between diameters. Next, we plotted TV12 vs the calculated surface area, but no qualitative improvement was observed. More important features differentiating targets of similar PTV could be the convex character of the target, as well as variation between dosimetrists.

Data for different PD values were generated from 50 clinically approved CAT plans, a corresponding 50 re‐plans using DCAT planning, and by re‐scaling the dose distribution after changing the PD values to 15, 18, 21, or 24 Gy. As discussed above, if only the PD is changed, then the relative spatial dose distribution is unchanged. It follows that COV and CI should be identical as should the volume enclosed by a specified (as a percentage of PD) isodose surface. Using this fact, we mostly avoided recalculating the dose, and just recorded TV12 for the desired PD by identifying the corresponding isodose percentage from the DVH. But it should be noted that if one performs the corresponding dose recalculations instead, the COV, CI, and TV12 values can be slightly different. In BrainScan, this difference occurs because the smallest unit of dose reported by the DVH is 1% of the PD. So, when the PD is changed, the irradiated volumes derived from the DVH can change accordingly. It was expected that these changes would not make any significant difference in the value of our pre‐planning tools. We tested this by first re‐calculating the dose distribution for 34 plans (17 CAT plans and the corresponding 17 DCAT re‐plans) for which the PD was changed from 24 to 21 Gy. Then, we recorded the values of COV, CI, and TV12 from the recalculated DVH (PD = 21 Gy), and compared them to those values determined from the initial DVH (PD = 24 Gy). For the TV12 comparison, we recorded TV50% in the recalculated DVH so that both values correspond to TV12 for PD = 24 Gy. For COV, the number of recalculations resulting in no change was 24 while the maximum change was 0.09%. The corresponding values for CI and TV12 were (28, 0.02) and (21, 0.01 cc), respectively. Changes of this magnitude would have no discernable effect on the TV12 model, or clinical consequence. Thus, we could confidently collect TV12 data for different PD values without having to recalculate the DVH. Although it is important to state that generating TV12 data by scaling a clinically delivered plan to a higher PD is not an endorsement to treat an identical plan with a higher PD, but rather a quick and accurate way to extend our tool's range of applicability so that physicians can avoid specifying a PTV, PD pairing that cannot be achieved with an acceptable V12.

**Figure 6:**
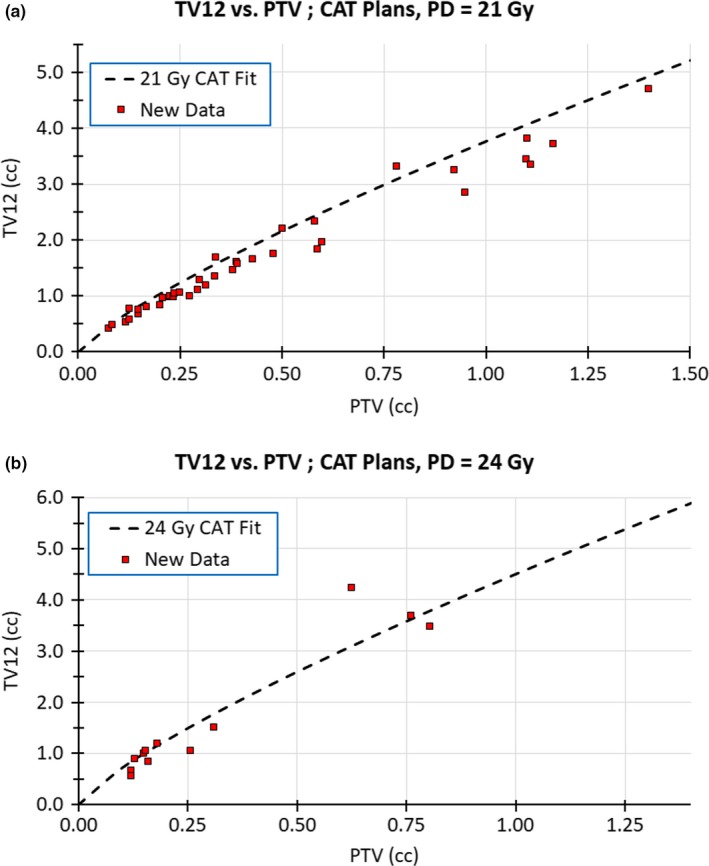
Model fit of this manuscript overlaid with new data. New data were collected from 50 isocenters in clinically approved plans treated after the dates used for the model fitting of this manuscript. Due to the distribution of plan type and dose prescription treated in our clinic, the new data presented are from circular arc therapy (CAT) plans with a prescription dose (PD) of either 21 or 24 Gy. The new data were overlaid on this manuscript's model fit for the total volume receiving at least 12 Gy (TV12) as a function of the planning target volume (PTV) in subfigures (a) PD = 21 Gy and (b) PD = 24 Gy. These figures display good agreement between the new data and the model fit, thus showing that the model fit is a useful tool for pre‐plan forecasting of TV12. In subfigure (b), the outlying data point at (0.626, 4.232) was due to beam overlap in a multi‐iso plan. Our model used data only from single‐iso plans, and thus may underestimate TV12 values in multi‐iso plans.

Our results have been clinically useful. Once a radiation oncologist has completed contouring the GTV, and specified the margin, then the PTV is generated, and Fig. 2 is used to predict the expected TV12 for a given PD and plan type. From this, the expected V12 (=TV12‐GTV) is found. Next, a PD and plan type which yield an acceptable value for the expected V12 is selected. The radiation oncologist can also change the expected V12 by modifying the margin added to the GTV to achieve a different PTV. This pre‐planning procedure has enabled our clinic to consistently produce plans where the V12 is in the first risk quartile specified by Minniti et al.^6^ where the risk of necrosis is lowest. Data from patient plans treated after the dates used for the model fitting of this manuscript are plotted in Fig. 6. These data agree with the model fit and show that in most cases a lower TV12 was used for treatment. However, it must be emphasized that the model presented in this manuscript was generated using data from single‐isocenter plans where the TV12 volume enclosed brain parenchyma only. As a result, the model will overestimate TV12 for cases where the TV12 surface encloses material other than brain parenchyma, and can underestimate TV12 in multi‐isocenter plans where overlap of beams attached to different isocenters can contribute additional dose [see Fig. 6(b)].

### 4.2 Comparison of conformity and low‐dose coverage between CAT and DCAT plans

Our TV12 model is fit for both CAT and DCAT planning types. Figure 2 shows the fit curves for both plan types plotted on the same axis. We see that for a given PD value, each pair of fit curves (corresponding to CAT or DCAT plan types) can be characterized by the difference between TV12 values. In each case, there is a value of PTV below (above) which the TV12 value associated with DCAT (CAT) planning is expected to be the greater of the two. Using Eq. (5), we can always approximate expected values of TV12. However, this does not provide any information on the relationship between TV12 and CI, which can initially be seen as counterintuitive, and can lead to a suboptimal choice of plan type. Specifically, DCAT plans more often achieve greater conformality (smaller CI) but also greater low‐dose coverage (greater TV12). While an inexperienced practitioner might reasonably assume that a lower CI value implies a lesser TV12 value, and thus choose a plan type based on CI value, alone. An accurate intuition on TV12 and CI vs PD and PTV can be developed by studying Fig. 5, which presents a clearer picture of how CI and TV12 values depend on plan type, and how the size of these differences depends on PTV and PD. Though best practice is for an interested clinic to generate similar plots from their own data so that differences in planning procedure, dosimetric goals, and radiation delivery system are taken into account.

The sequence of plots in Fig. 5 shows that the TV12 is most usually increased in a DCAT re‐plan, while the CI is usually decreased. Each plot has a fixed value of PD (15, 18, 21, or 24 Gy), and while the CI does not change with change in PD, we see that an increase in PD results in an increase in both the number of plans with positive TV12 change, and the magnitude of those changes. We now provide intuitive explanations of these trends.

The conformality and TV12 differences can be explained by considering the beam collimation systems used to deliver each plan type: mMLCs (DCAT) and stereotactic cones (CAT). On the one hand, the mMLC system can achieve a far greater variety of aperture shapes than can stereotactic cones, which are limited to fixed diameter circular apertures. For this reason, DCAT plans almost always achieve greater conformality (lesser CI), which is especially desirable when the target is larger than the greatest cone diameter, is oddly shaped, or is adjacent to an OAR. While, on the other hand, the mMLC aperture is located further from the isocenter than that of the stereotactic cone, which means that the mMLC generates a greater geometric penumbra than does the stereotactic cone.^11^, ^22^ The larger penumbra is the main reason that the low‐dose coverage (TV12) is usually greater for DCAT plans.

This trend in TV12 difference as a function of PD can be explained by qualitative analysis of dose drop‐off characteristics general to treatment planning.^5^ We perform a simplified analysis by considering a typical dose distribution along a straight‐line path beginning at the isocenter. The essential point is that for a given dose distribution, if only the specified PD is changed, and the rest of the dose distribution is scaled by the same factor, then the absolute doses change, but the percent isodose lines stay the same. Since both CAT and DCAT plan doses can be scaled in this fashion, this point, and thus the following analysis, is true for both plan types. It follows that for different PD, the 12 Gy isodose line comes at a different percentage of the PD. If the prescription isodose line is fixed at 80% of the maximum, then for PD = 15, 18, 21, or 24 Gy, the percent isodose line for 12 Gy is 64, 53.3, 45.7, or 40%, respectively. Now, if only the PD is changed, then the relative dose distribution does not change, and neither does the distance between the positions of any two dose values relative to the maximum dose of the plan. So, when the PD is increased, the relative dose percentage corresponding to 12 Gy decreases, while the PIDL remains fixed. It follows that the distance between the PIDL and the 12 Gy isodose line increases as well, which leads to a larger value of TV12.

### 4.3 Relationship of our study to previously published work

There are several published studies featuring models of SRS plans that predict V12 or TV12.^4^, ^14^, ^19^, ^21^, ^23^, ^24^, ^25^, ^26^ Two of these focused on multi‐target plans, where inter‐target dose overlap can increase V12.^14^, ^24^ Thomas et al.^14^ compared multi‐target plans generated for Gamma Knife or VMAT delivery. They used their VMAT data to model V12 as a function of both total number of targets and total volume of targets, but in contrast to our study the prescription dose was fixed at 18 Gy, and no margin was added to the GTV. Saghal et al.^24^ used multi‐target Gamma Knife plan data to model V12, and then suggest dose guidelines for multi‐target treatment. Shiraishi et al.^21^ used VMAT plans (mostly single target) to generate a sophisticated model to determine realistic DVHs for pre‐plan analysis and automated evaluation of plan quality. But, while the aforementioned model is a powerful clinical tool, its development and implementation requires a far greater investment in time and expertise than for our model. Narayanasamy et al.^25^ used GK Perfexion and GammaPlan (v 10.1) to demonstrate that the total V12 was correlated far more strongly with the total target volume, than with the number, shape, or location of the lesions. Zhao et al.^26^ used DCAT plan data to model the likelihood of radiation necrosis based on a logistic probability model depending on V12, which they modeled as a function of PTV and PD. The other three studies, like our own, analyzed single target plans to eliminate the effect of inter‐target dose overlap.^2^, ^4^, ^19^, ^23^ One such study used Gamma Knife data to model the increase in V12 as margin was added to the GTV,^23^ whereas another provided evidence suggesting a near equivalence for spatial dose drop‐off rate between different treatment delivery methods.^4^ In the former case, a 3D convex hull algorithm is used to help determine the empirical parameter value, while in both cases, the range of target volumes considered is much broader than in our study, and thus does not describe the non‐linear TV12 vs PTV relationship that we find by focusing on relatively small values (PTV ≤ 2.43 cc).

The single‐target planning study of Bohoudi et al.^19^ is most closely related to our study in that it models V12 using a two‐step process where the V12 vs PTV data sets are fit, and then those fit parameters are themselves fit with respect to their dependence on PD. In contrast to our study, they focus on large target volumes (PTV > 4 cc) where both fits are linear, and their data are only from DCAT plans where each PTV is generated by adding 1 mm of margin to the GTV. An important feature of their study is that their model, which, like ours, is based on single target plans, was validated by comparison with data from multi‐target VMAT plans. This implies that our TV12 model for DCAT could be useful for pre‐planning multi‐target VMAT cases where the target volumes are in the non‐linear region of the TV12 vs PTV curve. Our study is unique in that it models TV12 for both CAT and DCAT planning types, focuses on the range of small target volumes where TV12 vs PTV is non‐linear, promotes analysis of different margin sizes, and also compares CAT and DCAT planning techniques in the context of differences in conformity (ΔCI) and low‐dose spread (ΔTV12).

## 5 CONCLUSIONS

One can use locally collected dosimetric data to generate a model of V12 based on the GTV, PTV, PD, and SRS delivery method. The prediction of V12 informs the pre‐planning process, which minimizes time spent on re‐plans and attempts to achieve unrealistic dosimetric goals. In the absence of access to more sophisticated pre‐planning tools, this model can be locally generated and implemented at relatively low cost with respect to time, money, and expertise. For a particular PTV, this model provides an immediate calculated estimation of the expected TV12 for a specified PD without having to construct a treatment plan. This permits making a clinical decision on whether to change the PD or to treat the patient with more than a single fraction to limit toxicity.

Furthermore, when an SRS plan using CAT is re‐planned for DCAT, one can almost always achieve greater conformality (ΔCI<0), but, somewhat counterintuitively, the corresponding TV12 is often increased (ΔTV12>0). As PD increases, the number of re‐plans with ΔTV12>0 increases, as does the magnitude of ΔTV12 for each case. In addition, ΔCI has no dependence on PD, while, as PTV increases, ΔTV12 decreases. Thus, with respect to minimizing the risk of radiation necrosis, the most conformal plan is not always the best plan.

## CONFLICT OF INTEREST

The authors declare no conflict of interest.
